# Maternal Adiposity, Milk Production and Removal, and Infant Milk Intake During Established Lactation

**DOI:** 10.3390/nu17233726

**Published:** 2025-11-27

**Authors:** Zoya Gridneva, Ashleigh H. Warden, Xuehua Jin, Jacki L. McEachran, Ching Tat Lai, Sharon L. Perrella, Donna T. Geddes

**Affiliations:** 1 School of Molecular Sciences, The University of Western Australia, Crawley, WA 6009, Australia; ashleigh.warden@uwa.edu.au (A.H.W.); xuehua.jin@research.uwa.edu.au (X.J.); jacki.mceachran@uwa.edu.au (J.L.M.); ching-tat.lai@uwa.edu.au (C.T.L.); sharon.perrella@uwa.edu.au (S.L.P.); donna.geddes@uwa.edu.au (D.T.G.); 2 ABREAST Network, Perth, WA 6000, Australia; 3 UWA Centre for Human Lactation Research and Translation, Crawley, WA 6009, Australia

**Keywords:** breastfeeding, lactation, milk production, infant breast milk intake, adiposity, body composition, breast storage capacity, percentage of available milk removed

## Abstract

Background: Whilst maternal body mass index (BMI) is linked to suboptimal breastfeeding outcomes, maternal body composition has not been assessed with respect to milk production (MP). Methods: Lactating mothers 1–6 months postpartum (*n* = 281) completed a demographic questionnaire and a 24 h MP measurement using the test-weigh method, enabling the calculation of 24 h MP parameters, breast storage capacity (BSC) and the percentage of available milk removed (PAMR). Body composition was measured with bioimpedance spectroscopy. Linear regression models were used to determine maternal and infant factors associated with MP parameters; structural equation modelling was used to assess the mediating role of BSC. Results: Higher maternal adiposity was associated with lower BSC (*p* ≤ 0.028), MP (*p* ≤ 0.003), infant breast milk intake (*p* ≤ 0.003) and total milk intake (*p* ≤ 0.026). Higher BSC was associated with higher MP (*p* < 0.001), with BSC confirmed as a mediator of the relationship between adiposity and MP (67.5%). Mean PAMR was negatively associated with BSC and milk removal frequency (both *p* < 0.001), and was lower in occasionally pumping compared to breastfeeding only (*p* = 0.037) and exclusively pumping mothers (*p* = 0.012). Conclusions: Our findings confirm maternal adiposity as a major contributor to low MP and reveal BSC, which is a measure of glandular tissue volume or breast development, as a mediator between adiposity and MP. This provides a rationale for antenatal lactation assessment of mothers and timely intervention in high-risk mothers to ensure they reach their full lactation potential.

## 1. Introduction

Breastfeeding duration and exclusivity are linked to multiple health benefits for lactating mothers and infants [1,2], including a reduction in obesity risk [3,4,5]. Low milk supply (LMS; <600 g/24 h, determined a priori based on published data for exclusively breastfed infants [6,7] and universally accepted) is one of the major reasons for early cessation of breastfeeding [8,9]. Understanding which modifiable and non-modifiable factors predispose women to LMS [10,11] will inform the development of interventions aimed at improving milk production (MP).

Animal obesity models show an impairment of lactation performance via induction of prolactin resistance [12], but it is unknown if this mechanism is directly translatable to lactating women. In humans, maternal obesity has been linked to reduced breastfeeding initiation rates [13,14,15,16,17], later onset of secretory activation [15,16,18,19,20], increased breastfeeding difficulties [21] and diminished breastfeeding confidence [22] followed by early introduction of commercial milk formula [19] and shorter durations of exclusive or any breastfeeding [13,14,15,16,17,18,23]. Rather than measuring maternal body composition (BC), previous studies have mostly focused on relationships between maternal body mass index (BMI) and MP, as BMI data are relatively easy to acquire, yet they poorly represent adiposity and the ratio of fat to lean tissue [24]. Further, BC provides a more accurate estimate of maternal metabolic and endocrine health, which directly influences pathways that regulate milk synthesis [25,26].

A recent systematic review has confirmed that higher maternal BMI/adiposity is related to delayed secretory activation [27]. However, the review identified only six studies that analysed maternal BMI, and MP was estimated as a volume of milk expressed at a single/some/all feeds over 24 h period, usually early in lactation. Half of the studies reported inverse associations between BMI and MP, and half failed to show any relationships. No studies were identified that measured maternal BC in relation to MP. Further, the meta-regression analysis of 58 papers that investigated associations between both maternal postpartum BMI (*n* = 28) and percentage fat mass (%FM) (*n* = 30) and infant 24 h milk intake, the proxy measure of MP, showed no relationships, which could be due to the methodology. The review highlighted methodological heterogeneity in acquiring the BMI/BC data, such as using pre-pregnancy BMI, and the lack of variability in adiposity, particularly at the higher end of the BMI range [27]. However, a recent cross-sectional study (*n* = 609) published after this review established that a ten-unit increase in maternal BMI indeed was associated with an 84 (−136, −32) mL/24 h decrease in MP during established lactation [28]. Another recent small study (*n* = 34), which used a correlation networks approach, linked higher maternal adiposity to low MP along with minimal breast growth during pregnancy and low mammary glandular tissue representation [29]. These mixed findings indicate that there is a need for sound research on relationships between maternal factors, including BC, and 24 h MP, which will help to establish mechanisms and pathways implicated in obesity-related LMS.

Effective milk removal is essential to the establishment and maintenance of MP, yet it is not known if maternal BC affects the mechanics of milk removal. The strength of both infant sucking and pump vacuums, has previously been shown to be related to increased milk removal [30]. However, it is hypothesised that larger breasts, which typically contain more adipose tissue, may impact an infant’s latch, decreasing sucking intensity and subsequently reducing prolactin release, thereby impeding MP [19,31]. Currently, this theory has yet to be substantiated. However, women struggling with breastfeeding often express milk with a breast pump to initiate lactation [32]. In cases of low MP, it may be perceived that the breast pump is not emptying the breast adequately; however, it is not known if maternal adiposity physically affects milk removal with a breast pump.

This study aimed to elucidate associations between maternal and infant factors, including maternal BC, 24 h MP, infant milk intake and milk removal parameters during established lactation to gain knowledge for potential interventions that may improve breastfeeding success.

## 2. Materials and Methods

### 2.1. Participants and Study Design

This is a cross-sectional study of lactating mothers from the Perth metropolitan area, Western Australia. Mothers participating in multiple ongoing projects attended a BC study visit at The University of Western Australia between April 2013 and January 2015, and August 2018 and May 2025. At the study visit, maternal BC was measured with bioimpedance spectroscopy. Mothers completed a demographic questionnaire. Either prior to or after the BC study visit, mothers conducted 24 h MP measurements at their home with electronic scales to enable calculation of 24 h MP parameters.

Selection criteria included mothers of singleton term-born infants with a birth weight of ≥2500 g between 1 and 6 months postpartum. Mothers of small-for-gestational-age infants were excluded as their MP may be compromised, particularly in those with co-occurring conditions, i.e., placental insufficiency and gestational diabetes mellitus (GDM) [33]. Participants who had breast reduction surgery were excluded from the study, as well as those with GDM and pre-existing diabetes, as diabetes may impact MP [34] and breastfeeding duration [35,36] via disruption of secretory activation through hormonal dysregulation [37].

This study was conducted in accordance with the Declaration of Helsinki and was approved by The University of Western Australia Human Research Ethics Committee (RA/4/20/4023, RA/4/20/5657, 2019/RA/4/20/6134, and 2019/RA/4/20/6407). Informed written consent was obtained from all participants.

### 2.2. Anthropometric and Body Composition Measurements

Mothers’ body weights were measured at 3.8 ± 1.1 (mean ± standard deviation (SD)) months postpartum using an electronic scale (accuracy ± 0.1 kg; Seca, Chino, CA, USA); height was self-reported by the majority of mothers or measured against a calibrated marked wall (accuracy ± 0.1 cm); BMI was calculated as kg/m^2^. Breast volume (cm^3^) was based on both the current bra cup size and band size reported by mothers, and calculated using an online chart [38]. Breast volume is a crude calculation, as there are likely some design differences between bra manufacturers; however, an accurate breast volume measurement is difficult to achieve [39].

Maternal BC was measured with bioelectrical impedance spectroscopy using an ImpediMed SFB7 bioelectrical impedance analyser (ImpediMed, Brisbane, QLD, Australia) according to protocols described previously [40] and applying resistivity coefficients for the healthy female [41]. Measurements were usually conducted between 10:00 am and 2:00 pm, and after a breastfeed/breast expression, although the volumes of milk removed do not make a significant difference, even to the BC measurements of the infants that consumed it [42]. As well as standard BC measurements (fat-free mass (FFM), fat mass (FM), and %FM), the height-normalised BC indices (FFMI, FMI) [43] and FM to FFM ratio (FM/FFM) were calculated.

### 2.3. 24 h Milk Profile Data Collection

The 24 h MP measurements were performed at 3.4 ± 1.0 months postpartum and 1.8 ± 3.8 weeks prior to the BC study visit using the gold-standard test-weighing method [7,44]. Briefly, pre- and post-feed weights of infants as well as weights of milk collection containers before and after breast expressions were recorded using electronic scales (±2.0 g; Electronic Baby Weigh Scale, Medela Inc., McHenry, IL, USA). Data for all feeds/expressions in one 24 h period, plus one feed/expression, were recorded and reported in grams [45]. Total infant milk intake was summed from the intake of breast milk, including breastfeeding and expressed breast milk (EBM) intake, and any fed commercial milk formula. Milk removal frequency (summed breastfeeding and breast expression frequencies) was recorded from the 24 h MP data.

The 24 h MP (g) was calculated using the formula below (Equation (1)), where *v_i_* is the volume of each breastfeed/expression, *N* is the total number of breastfeeds and expressions, and *T* is the elapsed time from the end of the first breastfeed/expression until the end of the last breastfeed/expression:

(1) $$MP=\sum_{i=2}^{N}v_{i}\frac{24}{T}$$

No correction for infant-related insensible water loss was made; thus, 24 h MP may be underestimated by approximately 10% [46]. During the 24 h MP measurement majority of mothers (*n* = 201) collected small (<2.0 mL) milk samples pre- and post-expressions and/or breastfeeds by manual expression into 5 mL polypropylene tubes (P5016SL, Techno Plas Pty Ltd., St Marys, SA, Australia). Samples were stored in the mother’s home freezer until transported to the laboratory, where fat concentration was analysed using the creamatocrit method [47].

**Figure 1:**
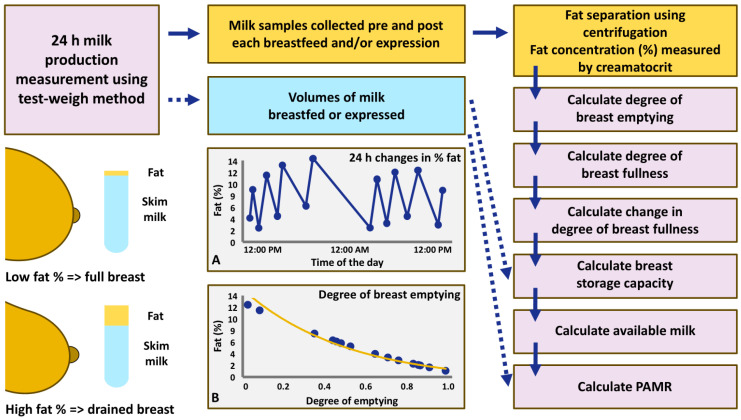
Flow diagram of determination of the breast storage capacity and percentage of available milk removed (PAMR) from the data collected during 24 h milk production measurement as described previously [46,48,49]. The solid dark blue arrows show the flow/use of the fat concentration data in the series of calculations, and the dotted dark blue arrows show the flow/use of the milk volumes data. The plots are examples of (**A**) changes in fat concentration (%) from pre-feed/expression to post-feed/expression during 24 h and (**B**) estimations of the degree of breast emptying based on the fat concentration (%).

Using the pre- and post-feed/expression fat concentrations and mothers’ 24 h MP data, the degree of fullness of the breast (the relative amount of milk stored in the breast at any one point in time) as well as the breast storage capacity (BSC, estimated from the amount of milk available to be removed when the breast is full) were calculated for each breast as described previously [46,48,49]. Briefly, there is a relationship between the milk fat concentration and the degree of fullness of the breast. Therefore, measuring the fat concentration changes from pre- to post-milk removal allows for calculation of the corresponding changes in the degree of fullness of the breast (Figure 1). The degree of fullness is calculated as 1 minus degree of emptying. These changes, together with milk volumes removed, enable the BSC calculation. From the 24 h MP data it was determined when the breast was most full and when it was most drained over the course of 24 h. The BSC of each breast was determined using a regression line relating the change in the degree of fullness at each feed/expression to the amount of milk removed from the breast during that feed/expression [46,48,49] and averaged for the analysis (mean BSC).

Knowing the calculated degree of fullness of the breast prior to each milk removal session, the percentage of available milk removed (PAMR; breast drainage) during 24 h was determined by dividing the volume of milk removed by the amount of milk available at the beginning of a breastfeeding or pumping session. Occasionally, when the actual milk volume removed surpasses the calculated available milk at the beginning of the session, PAMR may exceed 100%; the PAMR results were not capped where the calculated value exceeded 100%. The PAMR results during 24 h were averaged for each breast and averaged for the analysis (pooled mean PAMR).

### 2.4. Statistical Analyses

Using α = 0.05, recruitment of over 80 participants gave the study power of 0.80 to detect a small effect size (Cohen’s *f*^2^ below 0.1) [50]. Descriptive statistics are reported as mean ± SD and min–max for continuous variables and frequencies/counts and percentages for categorical variables, modelling results as parameter estimates (PE) ± standard errors (SE).

Statistical analysis was performed using the R-software package (version 4.3.2; R Foundation for Statistical Computing, Vienna, Austria). The R-packages nlme (version 3.1–96), multcomp [51], ggplot2 [52] and lavaan [53] were used for linear models, Tukey’s all-pair comparisons, structural equation modelling (SEM), data exploration and graphics, respectively. Statistical analysis used univariable linear models as well as univariable linear models accounting for infant birth weight for variables that had associations with it (the 24 h MP, mean BSC and infant milk intake variables). Where there were more than two levels of a categorical variable, Tukey’s all-pair comparisons were used to determine which levels differed.

Subsets by MP (LMS and normal milk supply) were used to investigate the associations between 24 h MP and milk removal frequencies (post hoc analysis). The 24 h MP, milk removal frequencies and infant milk intake were also investigated post hoc regarding participants’ expressing status (breastfeeding only, exclusively pumping, occasionally pumping (those expressed in total less milk than breastfed in 24 h) and predominantly pumping (those expressed in total more milk than breastfed in 24 h).

Following the univariable linear models, we further used SEM in an exploratory framework to examine the mediating role of BSC in the relationship between maternal adiposity (FM/FFM ratio, the key adiposity indicator as it showed the strongest association with 24 h MP in regression analyses) and 24 h MP. Infant birth weight was included as a covariate associated with 24 h MP. Milk removal frequency was modelled as an outcome of BSC, PAMR, parity and time postpartum to account for factors influencing feeding behaviour. A residual covariance between 24 h MP and milk removal frequency was included to capture shared variance not explained by the predictors, without adjusting away the mediated effect of BSC. All variables were standardised prior to SEM. The model was fitted using the robust maximum likelihood estimator (MLR) with full information maximum likelihood (FIML) to handle missing data. Model fit was assessed using Comparative Fit Index (CFI), Tucker–Lewis Index (TLI), root mean squared error of approximation (RMSEA), and standardised root mean squared residual (SRMR). The CFI and TLI > 0.90, and RMSEA and SRMR < 0.08 indicated a good fit [54] and, together with the absence of latent constructs and a ratio of approximately 8 observations per parameter, a sufficient sample size [55]. SEM results are reported as PE ± SE.

The significance level in this investigative study was set at *p* < 0.05. Missing data were addressed using available case analysis to avoid bias and loss of precision [56].

## 3. Results

### 3.1. Participants’ Demographics and Body Composition

Of the 286 lactating women who had completed both BC and 24 h MP measurements within 1–6 months postpartum, 1 participant was excluded due to having a genetic condition impeding their growth in childhood, and 4 due to the lack of data and/or data entry errors during 24 h MP measurements. The data of 281 participants were used in this analysis. Most participants identified as Australian (90%) and had a tertiary degree (69%) (Table 1). Just over half of the participants (55%) were primiparous, and 50% had a vaginal birth; all the infants were born at term. Higher birth weight was observed in male infants (0.208 ± 0.050 kg; *p* < 0.001) and infants with higher birth gestation (0.149 ± 0.022 kg; *p* < 0.001).

More than half of participants had overweight or obesity, 57% according to BMI category and 77% according to %FM [57] (Table 2).

### 3.2. 24 h Milk Production Parameters, Breast Storage Capacity and PAMR

**Figure 2:**
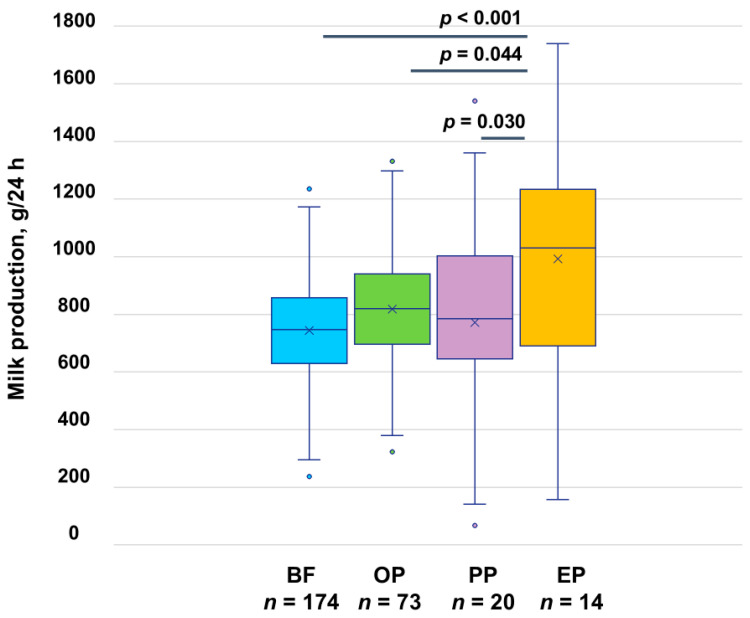
The 24 h milk production by participants’ expressing status. BF, breastfeeding only; EP, exclusively pumping; OP, occasionally pumping; PP, predominantly pumping. *p*-values indicate significant differences between exclusively pumping and other groups using Tukey’s all-pair comparisons.

The mean 24 h MP was 778 ± 238 g (Table 3); 50 mothers (17.8%) had LMS defined as <600 g/24 h [6,7], with 28% of them (*n* = 14) not supplementing with commercial milk formula during the 24 h measurement period. The 24 h MP in the LMS group was 446 ± 136 g, and in the normal milk supply group was 849 ± 189 g. Out of 279 infants, 24 (8.6%) had total milk intake <600 g. Among mothers, 61.9% (*n* = 174) breastfed only, 26.0% (*n* = 73) occasionally pumped, 7.1% (*n* = 20) predominantly pumped, and 5.0% (*n* = 14) exclusively pumped during the 24 h MP measurements (Figure 2).

The calculated mean BSC and pooled mean PAMR are presented in Table 3. The detailed 24 h MP and pooled mean PAMR by the participants’ expressing status are presented in Table 4 and Figure 2 (24 h MP only).

### 3.3. Milk Production

**Figure 3:**
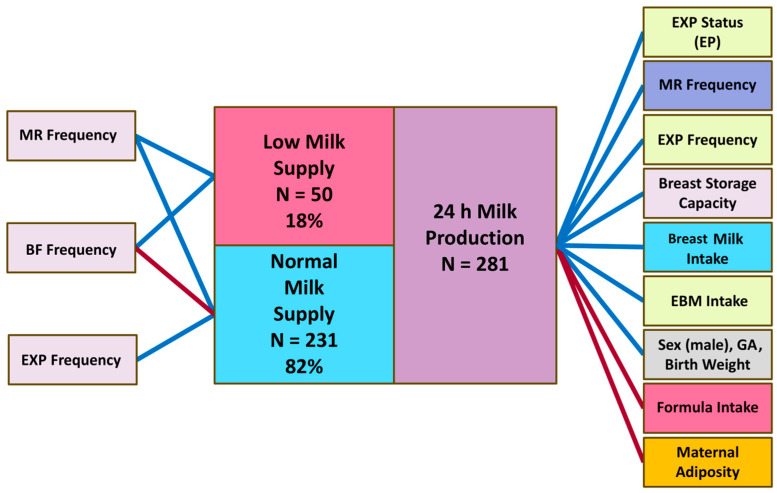
Significant associations of the 24 h milk production. BF, breastfeeding; EBM, expressed breast milk; EP, exclusively pumping; EXP, expressing; GA, gestational age; MR, milk removal. Blue lines indicate positive associations, red—negative associations.

The 24 h MP was higher in mothers of infants with higher birth weight (*p* = 0.002) and of male infants (*p* = 0.006), higher in those feeding more EBM and breast milk (including EBM), and lower in mothers supplementing with commercial milk formula (*p* < 0.001 for all; see Table 5 and Figure 3). The 24 h MP was higher in mothers with higher mean BSC (*p* < 0.001) but was not associated with pooled mean PAMR (*p* = 0.70). Accounting for infant birth weight made most of the associations stronger.

Twenty-four-hour MP was lower in mothers with higher body weight (*p* = 0.030) and adiposity, including BMI (*p* = 0.007), FM (*p* = 0.003), %FM (*p* = 0.002), FMI (*p* = 0.001) and FM/FFM ratio (*p* < 0.001; Table 5).

In the whole sample, 24 h MP was higher with higher milk removal frequency (*p* < 0.001) and expressing frequency (*p* < 0.001), but not with the breastfeeding frequency (*p* = 0.81; Table 5). However, the results differed by LMS and normal milk supply subgroups. After accounting for infant birth weight, in the normal milk supply subgroup, 24 h MP was higher with both higher milk removal frequency (7.98 ± 3.29 g, *p* = 0.016) and expressing frequency (21.93 ± 3.16 g, *p* < 0.001), yet the association with breastfeeding frequency was negative (−9.84 ± 2.93 g, *p* < 0.001). In the LMS subgroup, 24 h MP was higher with both higher milk removal frequency (9.72 ± 4.27 g, *p* = 0.027) and breastfeeding frequency (12.95 ± 3.76 g, *p* = 0.001), but not with expressing frequency (−8.87 ± 5.80, *p* = 0.13).

Expressing status was significantly associated with 24 h MP, with exclusively pumping mothers having higher MP compared with breastfeeding only (*p* < 0.001), occasionally pumping (*p* = 0.044) and predominantly pumping mothers (*p* = 0.030) (Table 5, Figure 2).

### 3.4. Infant Milk Intake

**Figure 4:**
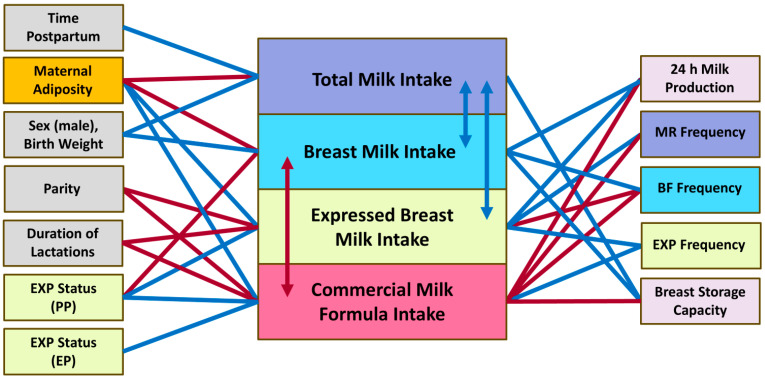
Significant associations of the infant milk intake. BF, breastfeeding; EP, exclusively pumping; EXP, expressing; MR, milk removal; PP, predominantly pumping. Blue lines and arrows indicate positive associations, red—negative associations.

The associations of infant milk intake with maternal and infant factors and 24 h MP parameters are presented in Table A1 and Table A2 and in Figure 4. Infant total milk intake was higher with higher breast milk (0.62 ± 0.03; *p* < 0.001) and EBM (0.13 ± 0.05; *p* = 0.031) intakes. The breast milk intake was lower with higher commercial milk formula intake (−0.95 ± 0.08; *p* < 0.001).

#### 3.4.1. Breast Milk Intake

The breast milk intake was higher in infants with higher birth weight (*p* < 0.001) and in male infants (*p* = 0.007), who were heavier at birth (Section 3.1). The breast milk intake was also higher in infants of mothers with higher breastfeeding frequency (*p* = 0.002), mean BSC (*p* < 0.001) and 24 h MP (*p* < 0.001; Table A1 and Figure 4). The breast milk intake was lower in infants of mothers with higher adiposity, including BMI (*p* = 0.005), FM (*p* = 0.003), %FM (*p* < 0.001), FMI (*p* < 0.001) and FM/FFM ratio (*p* < 0.001). Accounting for infant birth weight made most of the associations stronger. Expressing status was also associated with the breast milk intake, with infants of predominantly pumping mothers having lower breast milk intake compared with infants of breastfeeding only mothers (*p* = 0.043; Table A1 and Figure 4).

#### 3.4.2. Expressed Breast Milk Intake

The EBM intake was lower in infants of mothers with higher parity (*p* = 0.001), longer duration of previous lactations (*p* = 0.007) and higher breastfeeding frequency (*p* < 0.001), and was higher with higher expressing frequency (*p* < 0.001), milk removal frequency (*p* = 0.017) and 24 h MP (*p* < 0.001; Table A1 and Figure 4). The EBM intake was higher in infants of mothers with higher adiposity, including %FM (*p* = 0.048) and FM/FFM ratio (*p* = 0.043). Expressing status was also associated with the EBM intake (any expressing status, *p* < 0.001 for all) and progressive relationships between the pumping groups (*p* < 0.001 for all; Table A1 and Figure 4).

#### 3.4.3. Commercial Milk Formula Intake

The intake of commercial milk formula was lower in infants of mothers with higher parity (*p* = 0.016) and longer duration of previous lactations (*p* = 0.019) and higher in infants of mothers with higher expressing frequency (*p* = 0.002) and lower breastfeeding frequency (*p* < 0.001), milk removal frequency (*p* = 0.028), mean BSC (*p* < 0.001) and 24 h MP (*p* < 0.001; Table A2 and Figure 4). The intake of commercial milk formula was higher in infants of mothers with a higher FM/FFM ratio (*p* = 0.036). Expressing status was also associated with the intake of commercial milk formula, with infants of exclusively and predominantly pumping mothers having higher formula intake compared with infants of breastfeeding only or occasionally pumping mothers (*p* < 0.001 for all; Table A2 and Figure 4).

#### 3.4.4. Total Milk Intake

The total milk intake was higher in older infants (time postpartum: *p* = 0.038), in male infants (*p* < 0.001), in infants with higher birth weight (*p* < 0.001) and in infants of mothers with higher mean BSC (*p* < 0.001; Table A2, Figure 4). The total milk intake was lower in infants of mothers with higher body weight (*p* = 0.011) and BC parameters, including BMI (*p* < 0.001), FFMI (*p* = 0.026), FM (*p* = 0.002), %FM (*p* = 0.002), FMI (*p* < 0.001) and FM/FFM ratio (*p* = 0.001). Accounting for infant birth weight made most of the associations stronger. Expressing status was not associated with the total milk intake (*p* = 0.45; Table A2 and Figure 4).

### 3.5. Milk Removal Frequencies

#### 3.5.1. Breastfeeding Frequency

**Figure 5:**
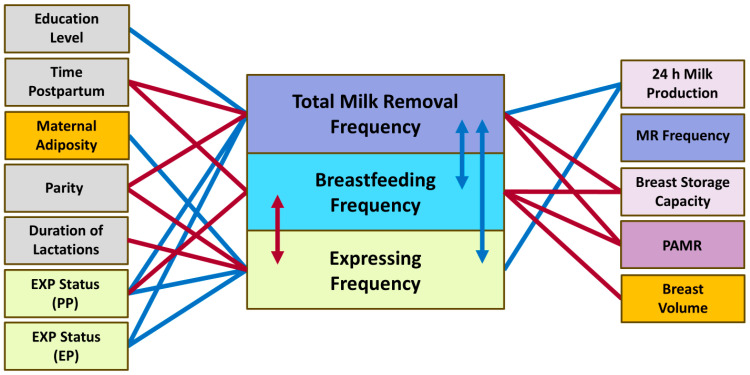
Significant associations of the milk removal frequencies. EP, exclusively pumping; EXP, expressing; MR, milk removal; PAMR, percentage of available milk removed; PP, predominantly pumping. Blue lines and arrows indicate positive associations, red—negative associations.

The associations of milk removal frequencies with maternal and infant factors and 24 h MP parameters are presented in Table 6 and Figure 5. The 24 h breastfeeding frequency declined with advancing postpartum time (*p* = 0.015) and was lower with higher expressing frequency (*p* < 0.001) and in mothers with higher breast volume (*p* = 0.007), mean BSC (*p* = 0.037) and pooled mean PAMR (*p* < 0.001). Maternal BC did not have any relationships with breastfeeding frequency; however, maternal height has shown positive association (*p* = 0.004; Table 6).

#### 3.5.2. Expressing Frequency

The 24 h expressing frequency was lower with higher breastfeeding frequency (*p* < 0.001), higher parity (*p* < 0.001), longer previous lactation duration (*p* = 0.004) and higher FFM (*p* = 0.047), and was higher with higher maternal adiposity, including %FM (*p* = 0.030) and FM/FFM ratio (*p* = 0.022; Table 6 and Figure 5).

#### 3.5.3. Milk Removal Frequency

The 24 h milk removal frequency was lower with later time postpartum (*p* = 0.001) and higher parity (*p* = 0.002) and in mothers with higher mean BSC (*p* < 0.001) and pooled mean PAMR (*p* < 0.001; Table 6 and Figure 5). The 24 h milk removal frequency was also lower in mothers with a lower education level compared with the tertiary educated mothers (ANOVA: *p* = 0.012; certificate/apprenticeship: −1.37 ± 0.53, *p* = 0.010; and high school: −1.78 ± 0.96, *p* = 0.064). The 24 h milk removal frequency was not different between exclusively pumping and both breastfeeding only (*p* = 1.00) and predominantly pumping (*p* = 0.18) mothers, as well as between predominantly pumping mothers and those who pumped occasionally (*p* = 0.56). However, occasionally pumping mothers had higher milk removal frequency than exclusively pumping (*p* = 0.003) and breastfeeding only mothers (*p* < 0.001); predominantly pumping mothers had higher milk removal frequency compared with breastfeeding only mothers (*p* = 0.013; Table 6 and Figure 5).

### 3.6. Breast Storage Capacity and PAMR

**Figure 6:**
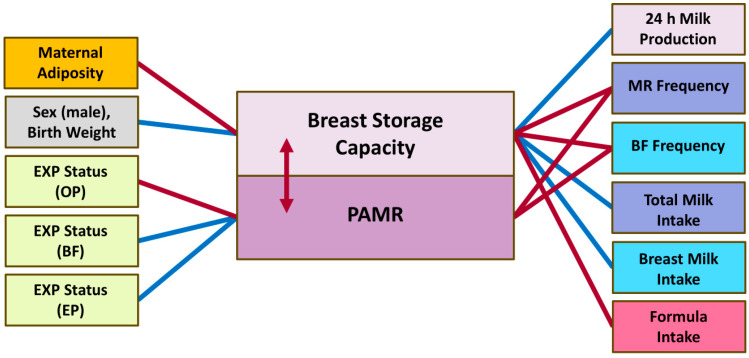
Significant associations of the breast storage capacity and PAMR. BF, breastfeeding; EP, exclusively pumping; EXP, expressing; MR, milk removal; OP, occasionally pumping; PAMR, percentage of available milk removed. Blue lines and arrows indicate positive associations, red—negative associations.

There were no significant differences between the left and right breast for both BSC (*p* = 0.36) and mean PAMR (*p* = 0.32). There was no association between the mean BSC and pooled mean PAMR (*p* = 0.24; Table 7); however, when assessed by the breast (*n* = 397), mean PAMR was lower when BSC was higher (left breast: −0.04 ± 0.02%, *p* = 0.023; right breast: −0.05 ± 0.02%, *p* = 0.006; and both breasts: −0.05 ± 0.01%, *p* < 0.001) (Figure 6).

The were no relationships between pooled mean PAMR and any infant or maternal factors, including maternal BC. The pooled mean PAMR was lower in mothers with higher breastfeeding frequency (−1.07 ± 0.20; *p* < 0.001) and milk removal frequency (−1.46 ± 0.21; *p* < 0.001). Participants’ expressing status was also associated with pooled mean PAMR (ANOVA *p* = 0.005), with occasionally pumping mothers having lower pooled mean PAMR compared with breastfeeding only (−5.37 ± 2.01; *p* = 0.037) and exclusively pumping mothers (−12.63 ± 4.10; *p* = 0.012), with no further difference between the other subgroups.

The mean BSC was higher with higher 24 h MP (*p* < 0.001; Table 5), in mothers of male infants (*p* = 0.007) and infants with higher birth weight (*p* = 0.004) and in those feeding more breast milk (*p* < 0.001) and total milk (*p* < 0.001) volumes. The mean BSC was lower in mothers with higher breastfeeding frequency (*p* = 0.037) and milk removal frequency (*p* < 0.001) and in those feeding more commercial milk formula (*p* < 0.001; Table 7 and Figure 6). The mean BSC was also lower in mothers with higher adiposity, including %FM (*p* = 0.002), FMI (*p* = 0.025) and FM/FFM ratio (*p* < 0.001). Accounting for infant birth weight made most of the associations stronger. Expressing status had no relationship with mean BSC (*p* = 0.98).

**Figure 7:**
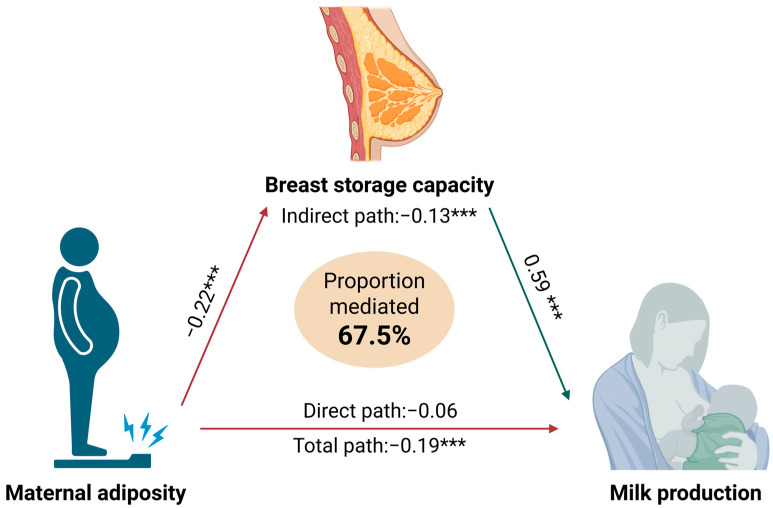
Breast storage capacity as a mediator of the relationship between maternal adiposity and milk production. All variables were standardised prior to analysis. Total path represents the change in 24 h milk production associated with a one-standard-deviation increase in the maternal fat mass-to-fat-free mass ratio. Indirect path represents the portion of this change that occurs through breast storage capacity. The direct path represents the portion of the change in 24 h milk production associated with maternal adiposity that is not mediated by breast storage capacity. Fit statistics for the model are shown in Table A3. *** *p* < 0.001. Created with www.BioRender.com.

Further, to examine whether the association between maternal adiposity and lower MP could be explained by the decreased BSC, we performed a mediation analysis using SEM. The model showed an acceptable fit to the data, with CFI = 0.98, TLI = 0.95, RMSEA = 0.05 and SRMR = 0.04 (Table A3). The maternal FM/FFM ratio was negatively associated with BSC (−0.22 (0.06), *p* < 0.001), and BSC was positively associated with 24 h MP (0.59 (0.07), *p* < 0.001). The direct effect of maternal adiposity on 24 h MP was not significant (−0.06 (0.04), *p* = 0.15), but the indirect effect through BSC was significant (−0.13 (0.04), *p* < 0.001), accounting for 67.5% of the total effect (Figure 7 and Table A3).

### 3.7. Breast Volume

The breast volume was higher with higher maternal BC (body weight: 12.55 ± 1.04; BMI: 39.42 ± 3.13; FFM: 121.62 ± 2.58; FFMI: 71.55 ± 8.21; FM: 19.06 ± 1.61; %FM: 23.64 ± 2.71; FMI: 55.54 ± 4.69; and FM/FFM ratio: 912.23 ± 102.80; all *p* < 0.001) and negatively associated with 24 h breastfeeding frequency (*p* = 0.007, Table 6). There were no significant associations between the breast volume and any other maternal or infant characteristics, as well as MP or milk removal parameters.

## 4. Discussion

**Figure 8:**
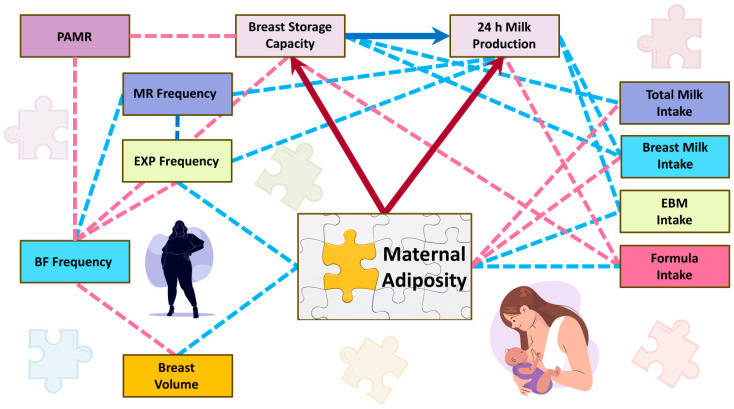
The findings of this study show that maternal adiposity may impede milk production and suggest that this relationship is mediated by breast storage capacity. Pink dotted lines and dark red arrows indicate negative relationships, and light blue dotted lines and the single dark blue arrow indicate positive relationships. BF, breastfeeding; EBM, expressed breast milk; EXP, expressing; MR, milk removal; PAMR, percentage of available milk removed.

This study has provided strong evidence that increased maternal adiposity may reduce MP in part by incomplete breast development measured as BSC (Figure 8). Increased maternal adiposity was also associated with less infant breast milk and total milk intakes, consistent with lower MP. Interestingly, the ability to remove milk from the breast was not related to maternal adiposity, casting doubt on latch issues being fully responsible for lower rates of breastfeeding in women with overweight or obesity.

### 4.1. Milk Production, Milk Intake and Maternal Adiposity

High maternal adiposity is not only a major risk factor for many chronic diseases, but also for pregnancy complications and reduced breastfeeding duration [17,58]. Still, the mechanisms leading to early cessation of breastfeeding are to be elucidated. This study provides strong evidence that increased maternal adiposity is associated with lower MP, likely mediated through reduced breast development. This is consistent with a recent ultrasound study reporting that higher maternal adiposity correlates with minimal pregnancy breast growth and low mammary glandular tissue representation during established lactation [29].

Maternal adiposity parameters, such as FM, FMI, %FM and FM/FFM ratio, were significantly associated with several 24 h MP measures, contrary to inconclusive relationships of MP with BMI [27], which is not representative of adiposity and a poor indicator of health [24]. Using bioimpedance spectroscopy, we identified 20% more women with adiposity above the normal range compared with BMI (Table 2). Whilst there are no reference data available for healthy postpartum BMI or %FM range, bioimpedance spectroscopy provides accurate adiposity measures and should be used in future studies. Importantly, increased adiposity measures are associated with lower breast development (BSC) and MP (Table 5 and Table 7). Interestingly, women with higher adiposity expressed more frequently, potentially as an effort to increase MP (Table 6). We also observed that higher maternal adiposity and BMI were associated with lower breast milk intakes and greater formula intakes (Table A1 and Table A2), likely reflecting reduced MP.

Around 9% of infants in our study had a total milk intake below 600 g, and under a third of participants with LMS did not supplement with formula. This may negatively affect infant growth if an infant receives inadequate nutrition over a period of time. As we only captured a 24 h period, it may not be reflective of the usual mothers’ practices. It also may be possible that our study population is biased in that participants with a strong intention to fully breastfeed and suffering LMS may be seeking information whilst not supplementing their infant or not supplementing adequately. Further, the amounts of milk and formula that infants of mothers with obesity receive are rarely measured, and these mothers may feed less formula to protect their infants from obesity and avoid adverse health outcomes [59,60]. Women with obesity may also feel judged, perceiving formula use as a choice rather than a necessity [61]. Regardless of these influences, routine nutritional screening of infants of mothers with LMS for adequacy of nutrition should be considered in clinical practice.

### 4.2. Breast Storage Capacity as a Mediator of Maternal Adiposity and Milk Production Relationships

We have found that the association of high maternal adiposity with low MP is mediated by lower breast development as measured by BSC, with the indirect effect through BSC accounting for 67.5% of the total effect (Figure 6). This is consistent with the positive relationship between BSC and 24 h MP reported in a smaller Australian study [39] in which this relationship was stable between 1 and 6 months postpartum.

BSC approximates the amount of milk-secreting glandular tissue in the breast. Ultrasound data show that BSC correlates positively with duct diameters and MP [29]. BSC was not correlated with breast size (Table 7), likely because higher adiposity increases breast volume predominantly through adipose rather than glandular tissue [62]. BSC, therefore, represents a more meaningful non-invasive estimate of glandular tissue volume than breast size [29,62].

### 4.3. Milk Removal Frequencies and Breast Storage Capacity

The capacity of the breast to store milk must impact the frequency of breastfeeding to some degree. For example, women with smaller BSC are more likely to need to feed more frequently than women who can store large volumes of milk. As such, women with LMS are likely have smaller BSC and would need to feed very frequently to meet their infants’ nutritional requirements [63]. This is supported by our observation that higher BSC was associated with lower milk removal frequency (Table 7). However, increasing milk removal frequency may not fully compensate for limited glandular capacity as milk synthesis is constrained by lactocyte activity [64]. Thus, women may be unable to overcome suboptimal glandular development solely through increased milk removal frequency [65].

Noteworthily, the 24 h milk removal frequency was 1.4 feed/expression lower in mothers with lower education, suggesting that education may be a mediator of the autocrine control of lactation rather than a direct cause of LMS; however, we do have a high representation of well-educated women. Thus, effort should be made to teach the health benefits and physiology of breastfeeding [66,67,68,69,70] at lower education levels, including at high or even primary school, which has been shown to improve breastfeeding in several countries [71,72].

### 4.4. Expressing Status and Milk Removal Efficacy

In line with more effective emptying of the breast, exclusively pumping mothers had higher PAMR and MP compared to all other groups (Table 5, Figure 1, Section 3.5). Indeed, it has been shown that exclusively/predominantly pumping mothers are able to sustain lactation if no intrinsic LMS issues are present [73]. The same study reported that milk ejection, milk removal efficacy and milk flow parameters were not different in predominantly pumping compared to breastfeeding only or occasionally pumping participants. Importantly, milk removal efficacy was unrelated to the volume of glandular tissue (BSC), indicating that low pumped volumes among women with LMS likely reflect reduced milk synthesis rather than pump efficiency (Section 3.5 and Table 7).

However, when assessed by individual breast, lower effectiveness (PAMR) was related to higher BSC (Section 3.5), expected for a predominantly breastfeeding population, in which infants fed on demand do not fully empty the breast, only consuming 67% of available milk on average [7]. The pooled mean PAMR was also lower in mothers with higher breastfeeding and total milk removal frequencies, but was not related to expressing frequency (Section 3.5). The expressing frequency was lower with higher parity, longer duration of previous lactations and higher maternal FFM, yet higher with increased maternal adiposity (%FM and FM/FFM; Table 6, Figure 4). The latter is expected as women with obesity are more likely to try pumping and may have a higher need for it in the early postpartum period [31,74] due to a delayed onset of secretory activation [15,16,18,19,20], again positioning maternal adiposity as a central factor impacting breastfeeding behaviour and MP on multiple levels.

Overall, PAMR was not associated with maternal adiposity or infant characteristics (Section 3.5 and Table 7), suggesting that while adiposity affects milk synthesis, it does not impair milk removal efficiency.

### 4.5. Infant and Maternal Characteristics

In our study, mothers of infants with higher birth weight, as well as mothers of male infants, had higher MP, and their infants had higher intake of breast milk and total milk intake, likely reflecting the higher energy needs of bigger infants (Table 5). These results are consistent with studies that reported higher infant milk intake in infants with higher birth weight [75,76] and in male infants [7,77,78,79]. The higher MP of mothers with heavier and male infants is also reflected in high glandular volume (BSC) (Table 5). This indicates that heavier infants may have an increased demand for milk, and this may result in some increase in proliferation of lactocytes in the transitional period to full production [80,81,82], and that reported infant sex relationships with MP and intake could be fully explained by the birth weight relationships.

We also found that mothers of higher parity and with longer duration of previous lactations were more likely to be breastfeeding only and not supplementing with EBM and formula (Table 6, Table A1 and Table A2). This is similar to a recent longitudinal study, which reported lower formula intake during consecutive lactations for infants of mothers who were partially breastfeeding during previous lactation [75]. This increase in MP is supported by evidence that higher parity and duration of previous lactations impact the number of the milk ducts and BSC is related to higher 24 h MP [29] and by animal studies that report greater milk yield with increasing parity [83,84,85]. Additionally, women are more likely to report LMS during their first lactation [9,86], with lower 24 h MP observed in primiparous compared to multiparous women during the first week postpartum [87,88]. However, several other studies have shown no relationship between parity and infant milk intake [7,89,90,91], potentially due to smaller sample sizes and heterogeneity in methodology, highlighting the need for larger longitudinal studies.

### 4.6. Strengths and Limitations

There are multiple strengths in our study, including a robust sample size, using validated reference methods, such as bioelectrical impedance spectroscopy for measuring maternal BC [92] and test-weighing for 24 h MP [93], as well as collecting pre- and post-milk removal milk samples, allowing the calculation of BSC and PAMR. Our sample consists of women during established lactation who delivered term infants, without GDM and pre-existing diabetes that present challenges and may affect MP [34,37], thus excluding several intrinsic reasons for LMS. Our participants also had a broad BMI range, with high representation of those with overweight and obesity (57%), one of the major limitations of other studies [27]. Limitations include the cross-sectional design and the predominantly highly educated, high-income population with mainly European ancestry, which may limit generalizability to more diverse settings. We also made no correction for infant-related insensible water loss; thus, 24 h MP may be underestimated [46].

## 5. Conclusions

Our findings corroborate animal studies showing that high adiposity impacts milk production. Further, we identified glandular breast volume (breast storage capacity) as a mediator of relationships between adiposity and milk production. These results provide a rationale for antenatal lactation assessment of women and timely intervention in high-risk mothers to ensure they reach their full lactation potential. The results also may inform future interventions, such as maintaining healthy adiposity. Encouragingly, maternal adiposity seems to contribute little to the efficacy of milk removal; thus, if small volumes are removed by the infant or pump, this may be reflective of low milk production. Therefore, improving milk synthesis should be the focus of potential interventions for improving breastfeeding outcomes.

## Figures and Tables

**Table 1:** Participants’ demographic characteristics.

Parameters	Mean ± SD or *n* (%) and Min–Max
Maternal characteristics	*n* = 281
Age (years)	32.7 ± 4.2 (23–44) ^1^
Parity (primiparous)	155 (55.2) (1–4)
Highest education level	*n* = 281
High school	17 (6.0)
Certificate/apprenticeship	71 (25.3)
Tertiary degree	193 (68.7)
Race	*n* = 278
Australian	251 (90.3)
Asian	21 (7.6)
Other	6 (2.1)
Infant characteristics	*n* = 281
Sex (Male)	139 (49.5)
Birth gestation (weeks)	39.5 ± 1.1 (37.0–43.0)
Birth weight (kg)	3.498 ± 433 (2.540–5.045)
Birth mode	*n* = 278
Spontaneous vaginal	138 (49.6)
Assisted vaginal	38 (13.7)
Elective caesarean	60 (21.6)
Non-elective caesarean	42 (15.1)

^1^ Data are mean ± standard deviation (SD) or *n* (%) and min–max where appropriate.

**Table 2:** Participants’ anthropometric and body composition characteristics.

Maternal Characteristics *(n* = 281)	Mean ± SD or *n* (%)	Min–Max
Weight (kg)	73.6 ± 15.1 ^1^	42.6–122.0 ^2^
Height (cm)	166.4 ± 6.9	143.0–185.0
Body mass index (kg/m^2^) (*n* = 281)		
Overall	26.5 ± 4.8	17.3–41.9
<18.5 kg/m^2^	7 (2.5)	17.3–18.4
18.5–24.9 kg/m^2^	114 (40.6)	18.8–24.9
25.0–29.9 kg/m^2^	95 (33.8)	25.0–29.9
>30 kg/m^2^	65 (23.1)	30.0–41.9
Fat-free mass (kg)	45.3 ± 6.9	29.9–74.8
Fat-free mass index (kg/m^2^)	16.3 ± 2.1	11.6–24.0
Fat mass (kg)	28.3 ± 9.6	10.2–58.4
Fat mass index (kg/m^2^)	10.2 ± 3.3	3.7–19.8
Fat mass (%) (*n* = 281)		
Overall	37.6 ± 6.3	19.2–53.3
<21.0%	2 (0.7)	19.2–20.4
21.0–32.9%	64 (22.8)	23.2–32.8
33.0–38.9%	94 (33.5)	33.0–38.9
>39%	121 (43.1)	39.0–53.4
Fat mass to fat-free mass ratio	0.56 ± 0.17	0.24–1.14
Breast volume (cm^3^) ^3^	780.8 ± 304.0	180–1580

^1^ Data are mean ± standard deviation (SD) and ^2^ min–max. ^3^ *n* = 243.

**Table 3:** The 24 h milk production and infant milk intake.

Parameters (*n* = 281)	Mean ± SD	Min–Max
Total 24 h milk production (g)	778 ± 238 ^1^	67–1739 ^2^
Milk removal frequency (per breast)	12.3 ± 3.8	4–25
Breastfeeding frequency (per breast)	10.3 ± 4.2	0–22
Milk intake from the breast (g)	672 ± 280	0–1344
Expressing frequency (per breast)	2.0 ± 3.5	0–16
Pumping session frequency	1.2 ± 2.1	0–11
Breast milk expressed (g)	131 ± 282	0–1870
Expressed breast milk intake (g) ^3^	86 ± 198	0–1200
Intake of breast milk (g) ^3,4^	763 ± 208	0–1344
Commercial milk formula intake (g) ^5^	42 ± 129	0–775
Total milk intake (g) ^3,6^	805 ± 167	348–1344
Mean breast storage capacity (g) ^7^	160.8 ± 55.3	36.3–354.9
Pooled mean PAMR (%) ^8^	74.3 ± 12.8	34.9–125.9

^1^ Data are mean ± standard deviation and ^2^ min–max. ^3^ *n* = 279. ^4^ Includes infant intake of breast milk from the breast and expressed breast milk. ^5^ *n* = 280. ^6^ Includes infant intake of breast milk from the breast, expressed breast milk and formula. ^7^ *n* = 201 and ^8^ *n* = 199.

**Table 4:** 24 h milk production, breast storage capacity and percentage of available milk removed by participants’ expressing status.

Parameters	Mean ± SD	Min–Max
All mothers	*n* = 281	
24 h milk production (g)	778 ± 238 ^1^	67–1739 ^2^
24 h milk breastfed (g)	672 ± 280	0–1344
24 h milk expressed (g)	131 ± 282	0–1870
Left breast storage capacity (g) ^3^	159.7 ± 64.3	24.0–404.1
Right breast storage capacity (g) ^3^	162.3 ± 70.4	32.5–416.6
Mean breast storage capacity (g) ^4^	160.8 ± 55.3	36.3–354.9
Left breast mean PAMR (%) ^5^	74.1 ± 16.6	39.1–167.4
Right breast mean PAMR (%) ^4^	74.9 ± 17.9	25.2–190.6
Pooled mean PAMR (%) ^6^	74.3 ± 12.8	34.9–125.9
Breastfeeding only mothers	*n* = 174	
24 h milk production (g)	744 ± 181	238–1236
24 h milk breastfed (g)	775 ± 188	230–1344
24 h milk expressed (g)	0.0 ± 0.0	NA
Left breast mean PAMR (%) ^7^	75.6 ± 17.5	39.1–167.4
Right breast mean PAMR (%) ^8^	76.9 ± 19.2	25.2–190.6
Pooled mean PAMR (%) ^7^	75.8 ± 13.2	34.9–125.9
Occasionally pumping mothers	*n* = 73	
24 h milk production (g)	818 ± 223	324–1331
24 h milk breastfed (g)	675 ± 215	266–1156
24 h milk expressed (g)	167 ± 94	15–448
Left breast mean PAMR (%) ^9^	69.4 ± 12.2	44.2–99.2
Right breast mean PAMR (%) ^10^	69.9 ± 16.2	26.8–135.3
Pooled mean PAMR (%) ^9^	70.0 ± 10.9	41.3–104.9
Predominantly pumping mothers	*n* = 20	
24 h milk production (g)	772 ± 406	67–1540
24 h milk breastfed (g)	236 ± 166	18–538
24 h milk expressed (g)	539 ± 284	48–1060
Left breast mean PAMR (%) ^11^	70.5 ± 18.2	47.5–118.9
Right breast mean PAMR (%) ^12^	72.6 ± 13.9	48.3–93.9
Pooled mean PAMR (%) ^12^	72.0 ± 13.7	51.0–96.6
Exclusively pumping mothers	*n* = 14	
24 h milk production (g)	993 ± 444	158–1739
24 h milk breastfed (g)	0.0 ± 0.0	NA
24 h milk expressed (g)	991 ± 502	173–1870
Left breast mean PAMR (%) ^13^	82.2 ± 11.4	62.2–99.2
Right breast mean PAMR (%) ^13^	83.9 ± 8.1	72.5–95.6
Pooled mean PAMR (%) ^13^	83.0 ± 8.4	69.2–91.8

^1^ Data are mean ± standard deviation and ^2^ min–max. PAMR, percentage of available milk removed. ^3^ *n* = 201; ^4^ *n* = 199; ^5^ *n* = 198; ^6^ *n* = 197; ^7^ *n* = 114; ^8^ *n* = 115; ^9^ *n* = 58; ^10^ *n* = 59; ^11^ *n* = 15; ^12^ *n* = 14; ^13^ *n* = 11.

**Table 5:** The 24 h milk production associations.

Predictors (*n* = 281)	Univariable Models		Adjusted Models		
	PE ± SE	Predictor *p*-Value	PE ± SE	Predictor *p*-Value	Birth Weight *p*-Value
Time postpartum (months)	19.58 ± 13.82 ^1^	0.16	22.09 ± 13.62 ^2^	0.11	0.001
Birth mode ^4^	NA	0.37	NA	0.35	0.001
Infant factors					
Infant birth gestation (weeks)	22.67 ± 13.03	0.083	NA	NA	NA
Infant birth weight (g)	101.42 ± 32.26	**0.002**	NA	NA	NA
Infant sex (male)	77.54 ± 28.03	**0.006**	NA	NA	NA
Maternal factors					
Maternal age (years)	−3.40 ± 3.39	0.32	2.0 ± 3.5	0.32	0.002
Education level	NA	0.64	NA	0.63	0.002
Parity	−10.16 ± 18.09	0.58	−16.33 ± 17.90	0.36	0.001
Duration of previous lactations (months)	1.49 ± 1.32	0.26	0.43 ± 1.34	0.75	0.003
Breast volume (cm^3^) ^5^	0.03 ± 0.05	0.62	0.02 ± 0.05	0.77	0.007
Height (cm)	2.05 ± 2.07	0.32	0.71 ± 2.09	0.73	0.003
Weight (kg)	−2.05 ± 0.94	**0.030**	−2.51 ± 0.93	**0.007**	<0.001
Body mass index (kg/m^2^)	−7.97 ± 2.91	**0.007**	−8.69 ± 2.87	**0.003**	<0.001
Fat-free mass (kg)	−1.19 ± 2.05	0.56	−2.83 ± 2.07	0.17	<0.001
Fat-free mass index (kg/m^2^)	−7.86 ± 6.75	0.25	−11.25 ± 6.70	0.095	<0.001
Fat mass (kg)	−4.37 ± 1.45	**0.003**	−4.65 ± 1.43	**0.001**	<0.001
Fat mass (%)	−7.06 ± 2.23	**0.002**	−6.68 ± 2.20	**0.003**	0.003
Fat mass index (kg/m^2^)	−14.05 ± 4.26	**0.001**	−14.26 ± 4.2	**<0.001**	0.001
Fat mass to fat-free mass ratio	−290.63 ± 85.18	**<0.001**	−275.96 ± 84.13	**0.001**	0.003
24 h milk production parameters					
Breast milk fed (g) ^6,7^	0.95 ± 0.04	**<0.001**	0.95 ± 0.04	**<0.001**	0.88
Expressed breast milk fed (g) ^6^	0.33 ± 0.07	**<0.001**	0.35 ± 0.07	**<0.001**	<0.001
Commercial milk formula fed (g) ^8^	−0.91 ± 0.09	**<0.001**	−0.90 ± 0.09	**<0.001**	0.004
Total milk fed (g) ^6,9^	0.93 ± 0.06	**<0.001**	0.92 ± 0.06	**<0.001**	0.33
Breastfeeding frequency (each breast)	−0.80 ± 3.37	0.81	−1.31 ± 3.32	0.69	0.002
Expressing frequency (each breast)	15.93 ± 3.94	**<0.001**	17.53 ± 3.87	**<0.001**	<0.001
Milk removal frequency (each breast)	12.41 ± 3.63	**<0.001**	12.98 ± 31.61	**<0.001**	<0.001
Mean breast storage capacity (g) ^10^	2.67 ± 0.24	**<0.001**	2.59 ± 0.24	**<0.001**	0.12
Pooled mean PAMR (%) ^11^	0.51 ± 1.33	0.70	0.34 ± 1.30	0.79	0.003
Expressing status					
Expressing status	NA	**<0.001**	NA	**<0.001**	<0.001
EP ^12^ vs. BF ^13^	248.90 ± 64.33 ^3^	**<0.001**	254.47 ± 63.19	**<0.001**	<0.001
OP ^14^ vs. BF ^13^	73.68 ± 32.29	0.096	74.71 ± 31.71	0.080	<0.001
PP ^15^ vs. BF ^13^	28.26 ± 54.67	0.95	47.39 ± 53.99	0.80	<0.001
OP ^14^ vs. EP ^12^	−175.22 ± 67.56	**0.044**	−179.76 ± 66.36	**0.033**	<0.001
PP ^15^ vs. EP ^12^	−220.64 ± 80.69	**0.030**	−207.08 ± 79.34	**0.042**	<0.001
PP ^15^ vs. OP ^14^	−45.42 ± 58.44	0.86	−27.31 ± 57.64	0.96	<0.001

Data are parameter estimate (PE) ± standard error of estimate (SE); effects of predictors taken from ^1^ univariable linear models and ^2^ linear models that accounted for birth weight and ^3^ Tukey’s all-pair comparisons. Bold font indicates significant result. ^4^ *n* = 278; ^5^ *n* = 243; ^6^ *n* = 279. ^7^ Includes infant intake of breast milk from the breast and expressed breast milk. ^8^ *n* = 280. ^9^ Includes infant intake of breast milk from the breast, expressed breast milk and formula. ^10^ *n* = 201; ^11^ *n* = 199; ^12^ *n* = 14; ^13^ *n* = 174; ^14^ *n* = 73; ^15^ *n* = 20. BF, breastfeeding only; EP, exclusively pumping; NA, not applicable; OP, occasionally pumping; PAMR, percentage of available milk removed; PP, predominantly pumping.

**Table 6:** Milk removal frequencies’ associations.

Predictors (*n* = 281)	24 h Breastfeeding Frequency		24 h Expressing Frequency		24 h Milk Removal Frequency	
	PE ± SE	Predictor *p*-Value	PE ± SE	Predictor *p*-Value	PE ± SE	Predictor *p*-Value
Time postpartum (months)	−0.60 ± 0.24 ^1^	**0.015**	−0.13 ± 0.21	0.51	−0.73 ± 0.22	**0.001**
Birth mode ^3^	NA	0.63	NA	0.064	NA	0.48
Infant factors						
Infant birth gestation (weeks)	0.03 ± 0.23	0.90	−0.08 ± 0.19	0.67	−0.05 ± 0.21	0.80
Infant birth weight (g)	0.47 ± 0.58	0.42	−0.90 ± 0.48	0.064	−0.42 ± 0.53	0.43
Infant sex (male)	−0.30 ± 0.50	0.55	0.46 ± 0.42	0.27	0.16 ± 0.46	0.72
Maternal factors						
Maternal age (years)	0.05 ± 0.06	0.42	−0.02 ± 0.05	0.64	0.03 ± 0.06	0.65
Education level	NA	0.70	NA	0.081	***	**0.012**
Parity	0.18 ± 0.32	0.59	−1.09 ± 0.26	**<0.001**	−0.91 ± 0.29	**0.002**
Duration of previous lactations (months)	0.03 ± 0.02	0.30	−0.06 ± 0.02	**0.004**	−0.03 ± 0.02	0.13
Breast volume (cm^3^) ^4^	−0.002 ± 0.001	**0.007**	0.001 ± 0.001	0.073	−0.001 ± 0.001	0.19
Height (cm)	0.10 ± 0.04	**0.004**	−0.06 ± 0.03	0.059	0.05 ± 0.03	0.16
Weight (kg)	−0.0002 ± 0.02	0.99	−0.004 ± 0.01	0.78	−0.004 ± 0.02	0.79
Body mass index (kg/m^2^)	−0.06 ± 0.05	0.25	0.02 ± 0.04	0.68	−0.04 ± 0.05	0.37
Fat-free mass (kg)	0.03 ± 0.04	0.39	−0.06 ± 0.03	**0.047**	−0.03 ± 0.03	0.39
Fat-free mass index (kg/m^2^)	−0.08 ± 0.12	0.50	−0.12 ± 0.10	0.23	−0.20 ± 0.11	0.063
Fat mass (kg)	−0.02 ± 0.03	0.53	0.02 ± 0.02	0.31	0.01 ± 0.02	0.81
Fat mass (%)	−0.04 ± 0.04	0.32	0.07 ± 0.03	**0.030**	0.03 ± 0.04	0.37
Fat mass index (kg/m^2^)	−0.10 ± 0.08	0.21	0.09 ± 0.06	0.16	−0.01 ± 0.07	0.93
Fat mass to fat-free mass ratio	−1.76 ± 1.54	0.25	2.94 ± 1.27	**0.022**	1.18 ± 1.40	0.40
24 h milk production parameters						
24 h milk production (g)	−0.0003 ± 0.001	0.81	0.004 ± 0.001	**<0.001**	0.003 ± 0.001	**<0.001**
Breastfeeding frequency ^5^	NA	NA	−0.43 ± 0.04	**<0.001**	0.57 ± 0.04	**<0.001**
Expressing frequency ^5^	−0.63 ± 0.06	**<0.001**	NA	NA	0.38 ± 0.06	**<0.001**
Milk removal frequency ^5^	0.69 ± 0.05	**<0.001**	0.32 ± 0.05	**<0.001**	NA	NA
Mean breast storage capacity (g) ^6^	−0.01 ± 0.01	**0.037**	−0.01 ± 0.01	0.20	−0.02 ± 0.01	**<0.001**
Pooled mean PAMR (%) ^7^	−0.12 ± 0.02	**<0.001**	−0.01 ± 0.02	0.57	−0.13 ± 0.02	**<0.001**
Expressing status						
Expressing status	NA	**<0.001**	NA	**<0.001**	NA	**<0.001**
EP ^8^ vs. BF ^9^	−11.21 ± 0.88 ^2^	**<0.001**	11.29 ± 0.43	**<0.001**	0.08 ± 0.97	1.00
OP ^10^ vs. BF ^9^	0.40 ± 0.44	0.79	3.22 ± 0.21	**<0.001**	3.62 ± 0.49	**<0.001**
PP ^11^ vs. BF ^9^	−5.81 ± 0.74	**<0.001**	8.30 ± 0.36	**<0.001**	2.49 ± 0.83	**0.013**
OP ^10^ vs. EP ^8^	11.60 ± 0.92	**<0.001**	−8.07 ± 0.45	**<0.001**	3.54 ± 1.02	**0.003**
PP ^11^ vs. EP ^8^	5.40 ± 1.10	**<0.001**	−2.99 ± 0.53	**<0.001**	2.41 ± 1.22	0.18
PP ^11^ vs. OP ^10^	−6.20 ± 0.80	**<0.001**	5.08 ± 0.39	**<0.001**	−1.12 ± 0.88	0.56

Data are parameter estimate (PE) ± standard error of estimate (SE); effects of predictors taken from ^1^ univariable linear models and ^2^ Tukey’s all-pair comparisons. Bold font indicates significant result. ^3^ *n* = 278; ^4^ *n* = 243. ^5^ Frequencies from both breasts combined. ^6^ *n* = 201; ^7^ *n* = 199; ^8^ *n* = 14; ^9^ *n* = 174; ^10^ *n* = 73; ^11^ *n* = 20. *** See text below for detailed results. BF, breastfeeding only; EP, exclusively pumping; NA, not applicable; OP, occasionally pumping; PP, predominantly pumping.

**Table 7:** Breast storage capacity associations.

Predictors (*n* = 281)	Univariable Models		Adjusted Models		
	PE ± SE	Predictor *p*-Value	PE ± SE	Predictor *p*-Value	Birth Weight *p*-Value
Time postpartum (months)	2.15 ± 3.60 ^1^	0.55	2.84 ± 3.53 ^2^	0.42	0.003
Birth mode ^4^	NA	0.54	NA	0.52	0.005
Infant factors					
Infant birth gestation (weeks)	3.28 ± 3.73	0.38	−0.55 ± 3.91	0.89	0.006
Infant birth weight (g)	27.41 ± 9.30	**0.004**	NA	NA	NA
Infant sex (male)	21.03 ± 7.71	**0.007**	17.33 ± 7.76	**0.027**	0.013
Maternal factors					
Maternal age (years)	0.05 ± 0.92	0.96	0.10 ± 091	0.92	0.004
Education level	NA	0.95	NA	0.95	0.004
Parity	0.56 ± 5.04	0.91	−1.61 ± 5.00	0.75	0.004
Duration of previous lactations (months)	0.39 ± 0.34	0.25	−0.03 ± 0.35	0.94	<0.001
Breast volume (cm^3^) ^5^	0.03 ± 0.01	0.061	0.02 ± 0.01	0.096	0.032
Height (cm)	1.01 ± 0.56	0.076	0.63 ± 0.58	0.28	0.011
Weight (kg)	−0.07 ± 0.26	0.79	−0.24 ± 0.26	0.37	0.003
Body mass index (kg/m^2^)	−0.79 ± 0.82	0.34	−1.09 ± 0.81	0.18	0.002
Fat-free mass (kg)	0.99 ± 0.55	0.071	0.59 ± 0.56	0.29	0.012
Fat-free mass index (kg/m^2^)	2.10 ± 1.80	0.24	1.20 ± 1.80	0.51	0.006
Fat mass (kg)	−0.75 ± 0.41	0.072	−0.90 ± 0.41	**0.028**	0.002
Fat mass (%)	−1.93 ± 0.62	**0.002**	−1.92 ± 0.61	**0.002**	0.003
Fat mass index (kg/m^2^)	−2.74 ± 1.21	**0.025**	−2.97 ± 1.19	**0.013**	0.002
Fat mass to fat-free mass ratio	−79.21 ± 23.64	**<0.001**	−78.12 ± 23.18	**<0.001**	0.003
24 h milk production parameters					
Breast milk fed (g) ^6,7^	0.17 ± 0.02	**<0.001**	0.17 ± 0.02	**<0.001**	0.38
Expressed breast milk fed (g) ^6^	−0.01 ± 0.02	0.72	−0.004 ± 0.02	0.82	0.006
Commercial milk formula fed (g) ^8^	−0.19 ± 0.03	**<0.001**	−0.18 ± 0.03	**<0.001**	0.018
Total milk fed (g) ^6,9^	0.15 ± 0.02	**<0.001**	0.14 ± 0.02	**<0.001**	0.10
Breastfeeding frequency (each breast)	−1.91 ± 0.91	**0.037**	−2.05 ± 0.89	**0.023**	0.002
Expressing frequency (each breast)	−1.36 ± 1.05	0.20	−0.96 ± 1.04	0.36	0.006
Milk removal frequency (each breast)	−3.64 ± 0.99	**<0.001**	−3.44 ± 0.98	**<0.001**	0.007
Pooled mean PAMR (%)	−0.36 ± 0.31	0.24	−0.40 ± 0.30	0.19	0.003
Expressing status ^10^	NA	0.89 ^3^	NA	0.98	0.004

Data are parameter estimate (PE) ± standard error of estimate (SE); effects of predictors taken from ^1^ univariable linear models and ^2^ linear models that accounted for birth weight and ^3^ Tukey’s all-pair comparisons. Bold font indicates significant result. ^4^ *n* = 278; ^5^ *n* = 243; ^6^ *n* = 279. ^7^ Includes infant intake of breast milk from the breast and expressed breast milk. ^8^ *n* = 280. ^9^ Includes infant intake of breast milk from the breast, expressed breast milk and formula. ^10^ Results are not presented if ANOVA *p*-values are not significant. NA, not applicable.

## Data Availability

The data presented in this study are available on request from the corresponding author due to ethical restrictions.

## Appendix A

**Table A1:** Infant 24 h milk intake associations—breast milk and expressed breast milk intakes.

	Breast Milk Intake ^4^					Expressed Breast Milk Intake				
	Univariable Models		Adjusted Models			Univariable Models		Adjusted Models		
Parameters (*n* = 281)	PE ± SE	Predictor *p*-Value	PE ± SE	Predictor *p*-Value	Birth Weight *p*-Value	PE ± SE	Predictor *p*-Value	PE ± SE	Predictor *p*-Value	Birth Weight *p*-Value
Time postpartum (months)	19.07 ± 12.15 ^1^	0.12	21.47 ± 11.91 ^2^	0.073	<0.001	5.53 ± 11.64 ^1^	0.64	4.77 ± 11.65 ^2^	0.68	0.25
Birth mode ^5^	NA	0.46	NA	0.44	<0.001	NA	0.31	NA	0.31	0.25
Infant factors										
Infant birth gestation (weeks)	17.23 ± 11.46	0.13	NA	NA	NA	−4.18 ± 10.98	0.70	NA	NA	NA
Infant birth weight (g)	98.30 ± 28.12	**<0.001**	NA	NA	NA	−32.58 ± 27.36	0.24	NA	NA	NA
Infant sex (male)	67.17 ± 24.59	**0.007**	NA	NA	NA	23.78 ± 23.75	0.32	NA	NA	NA
Maternal factors										
Maternal age (years)	−1.97 ± 2.97	0.51	−1.92 ± 2.91	0.51	<0.001	−2.39 ± 2.83	0.40	−2.41 ± 2.83	0.39	0.23
Education level	NA	0.89	NA	0.89	<0.001	NA	0.10	NA	0.10	0.31
Parity	11.65 ± 15.84	0.46	5.75 ± 15.64	0.71	<0.001	−48.15 ± 14.86	**0.001**	−46.73 ± 14.96	**0.002**	0.39
Duration of previous lactations (months)	1.98 ± 1.15	0.085	0.88 ± 1.16	0.45	<0.001	−3.00 ± 1.11	**0.007**	−2.85 ± 1.15	**0.014**	0.63
Breast volume (cm^3^) ^6^	−0.0004 ± 0.04	0.99	−0.01 ± 0.04	0.82	0.002	0.07 ± 0.04	0.085	0.08 ± 0.04	0.065	0.080
Height (cm)	3.37 ± 1.81	0.064	2.11 ± 1.82	0.25	0.002	−1.19 ± 1.74	0.49	−0.78 ± 1.78	0.66	0.29
Weight (kg)	−1.53 ± 0.82	0.064	−1.97 ± 0.81	**0.016**	<0.001	0.23 ± 0.79	0.77	0.38 ± 0.80	0.64	0.21
Body mass index (kg/m^2^)	−7.15 ± 2.55	**0.005**	−7.83 ± 2.49	**0.002**	<0.001	1.17 ± 2.46	0.64	1.39 ± 2.47	0.58	0.22
Fat-free mass (kg)	0.26 ± 1.79	0.88	−1.26 ± 1.81	0.49	<0.001	−1.84 ± 1.71	0.28	−1.43 ± 1.76	0.42	0.34
Fat-free mass index (kg/m^2^)	−5.12 ± 5.91	0.39	−8.32 ± 5.85	0.16	<0.001	−5.33 ± 5.64	0.35	−4.42 ± 5.70	0.44	0.29
Fat mass (kg)	−3.86 ± 1.27	**0.003**	−4.12 ± 1.24	**0.001**	<0.001	1.53 ± 1.23	0.21	1.62 ± 1.23	0.19	0.21
Fat mass (%)	−7.21 ± 1.94	**<0.001**	−6.83 ± 1.91	**<0.001**	<0.001	3.74 ± 1.88	**0.048**	3.62 ± 1.88	0.056	0.28
Fat mass index (kg/m^2^)	−13.41 ± 3.71	**<0.001**	−13.59 ± 3.63	**<0.001**	<0.001	4.79 ± 3.61	0.19	4.85 ± 3.61	0.18	0.23
Fat mass to fat-free mass ratio	−296.8 ± 73.9	**<0.001**	−282.5 ± 72.7	**<0.001**	<0.001	146.56 ± 72.03	**0.043**	141.98 ± 72.13	0.050	0.28
24 h milk production parameters										
24 h milk production (g)	0.75 ± 0.03	**<0.001**	0.74 ± 0.03	**<0.001**	0.086	0.24 ± 0.05	**<0.001**	0.26 ± 0.05	**<0.001**	0.032
Breastfeeding frequency ^7^	9.12 ± 2.96	**0.002**	8.54 ± 2.92	**0.004**	<0.001	−26.68 ± 2.39	**<0.001**	−26.57 ± 2.40	**<0.001**	0.44
Expressing frequency ^7^	−6.18 ± 3.61	0.088	−4.73 ± 3.57	0.19	0.001	47.85 ± 1.93	**<0.001**	48.07 ± 1.95	**<0.001**	0.36
Milk removal frequency ^7^	5.62 ± 3.23	0.083	6.15 ± 3.16	0.053	<0.001	7.37 ± 3.07	**0.017**	7.21 ± 3.07	**0.019**	0.28
Mean breast storage capacity (g) ^8^	2.23 ± 0.20	**<0.001**	2.14 ± 0.20	**<0.001**	0.029	−0.10 ± 0.29	0.72	−0.07 ± 0.30	0.82	0.54
Mean PAMR (%) ^9^	0.31 ± 1.10	0.78	0.18 ± 1.07	0.87	<0.001	0.63 ± 1.25	0.62	0.66 ± 1.25	0.60	0.50
Maternal expressing status										
Expressing status	NA	**0.037**	NA	**<0.001**	0.002	NA	**<0.001**	NA	**<0.001**	0.81
EP ^10^ vs. BF ^11^	−96.25 ± 61.39 ^3^	0.38	−87.57 ± 60.43	0.45	0.002	678.51 ± 32.70 ^3^	**<0.001**	678.85 ± 32.79	**<0.001**	0.81
OP ^12^ vs. BF ^11^	−16.57 ± 28.68	0.93	−15.69 ± 28.21	0.94	0.002	81.00 ± 15.28	**<0.001**	81.04 ± 15.30	**<0.001**	0.81
PP ^13^ vs. BF ^11^	−126.18 ± 48.56	**0.043**	−109.87 ± 48.03	0.094	0.002	412.93 ± 25.87	**<0.001**	413.58 ± 26.06	**<0.001**	0.81
OP ^12^ vs. EP ^10^	79.68 ± 64.07	0.58	71.88 ± 63.06	0.65	0.002	−597.51 ± 34.13	**<0.001**	−597.82 ± 34.21	**<0.001**	0.81
PP ^13^ vs. EP ^10^	−29.93 ± 75.10	0.98	−22.30 ± 73.90	0.99	0.002	−265.58 ± 40.01	**<0.001**	−265.28 ± 40.09	**<0.001**	0.81
PP ^13^ vs. OP ^12^	−109.62 ± 51.91	0.14	−94.18 ± 51.28	0.24	0.002	331.92 ± 27.65	**<0.001**	332.54 ± 27.82	**<0.001**	0.81

Data are parameter estimate (PE) ± standard error of estimate (SE); effects of predictors taken from ^1^ univariable linear models and ^2^ linear models that accounted for birth weight and ^3^ Tukey’s all-pair comparisons. Bold font indicates significant result. ^4^ Includes infant intake of breast milk from breast and expressed breast milk. ^5^ *n* = 278; ^6^ *n* = 243. ^7^ Frequencies from both breasts combined. ^8^ *n* = 201; ^9^ *n* = 199; ^10^ *n* = 14; ^11^ *n* = 174; ^12^ *n* = 73; ^13^ *n* = 20. BF, breastfeeding; EP, exclusively pumping; NA, not applicable; OP, occasionally pumping; PP, predominantly pumping.

**Table A2:** Infant 24 h milk intake associations—commercial milk formula and total milk intakes.

	Commercial Milk Formula Intake					Total Milk Intake ^4^				
	Univariable Models		Adjusted Models			Univariable Models		Adjusted Models		
Parameters (*n* = 281)	PE ± SE	Predictor *p*-Value	PE ± SE	Predictor *p*-Value	Birth Weight *p*-Value	PE ± SE	Predictor *p*-Value	PE ± SE	Predictor *p*-Value	Birth Weight *p*-Value
Time postpartum (months)	0.96 ± 7.56 ^1^	0.90	0.52 ± 7.57 ^2^	0.95	0.29	20.29 ± 9.74 ^1^	**0.038**	22.23 ± 9.55 ^2^	**0.021**	<0.001
Birth mode ^5^	NA	0.46	NA	0.46	0.32	NA	0.51	NA	0.49	<0.001
Infant factors										
Infant birth gestation (weeks)	−7.88 ± 7.12	0.27	NA	NA	NA	9.51 ± 9.24	0.30	NA	NA	NA
Infant birth weight (g)	−19.23 ± 17.86	0.28	NA	NA	NA	79.13 ± 22.62	**<0.001**	NA	NA	NA
Infant sex (male)	12.90 ± 15.47	0.41	NA	NA	NA	80.42 ± 19.46	**<0.001**	NA	NA	NA
Maternal factors										
Maternal age (years)	0.89 ± 1.85	0.63	0.88 ± 1.85	0.64	0.28	−1.11 ± 2.39	0.64	−1.06 ± 2.34	0.65	<0.001
Education level	NA	0.38	NA	0.38	0.33	NA	0.88	NA	0.87	<0.001
Parity	−23.79 ± 9.76	**0.016**	−22.90 ± 9.83	**0.021**	0.41	−12.33 ± 12.74	0.33	−17.36 ± 12.54	0.17	<0.001
Duration of previous lactations (months)	−1.73 ± 0.73	**0.019**	−1.62 ± 0.76	**0.034**	0.56	0.24 ± 0.90	0.79	−0.75 ± 0.91	0.41	<0.001
Breast volume (cm^3^) ^6^	−0.03 ± 0.03	0.34	−0.03 ± 0.03	0.36	0.62	−0.03 ± 0.04	0.45	−0.04 ± 0.03	0.30	<0.001
Height (cm)	−1.31 ± 1.13	0.25	−1.10 ± 1.15	0.34	0.40	2.01 ± 1.46	0.17	0.96 ± 1.47	0.51	0.001
Weight (kg)	−0.16 ± 0.52	0.75	−0.08 ± 0.52	0.87	0.30	−1.69 ± 0.66	**0.011**	−2.06 ± 0.65	**0.002**	<0.001
Body mass index (kg/m^2^)	0.04 ± 1.61	0.98	0.17 ± 1.61	0.92	0.28	−7.09 ± 2.03	**<0.001**	−7.64 ± 1.99	**<0.001**	<0.001
Fat-free mass (kg)	−2.03 ± 1.11	0.069	−1.85 ± 1.14	0.11	0.51	−1.78 ± 1.44	0.22	−3.12 ± 1.44	**0.032**	<0.001
Fat-free mass index (kg/m^2^)	−5.45 ± 3.67	0.14	−4.97 ± 3.71	0.18	0.39	−10.53 ± 4.72	**0.026**	−13.26 ± 4.65	**0.005**	<0.001
Fat mass (kg)	0.67 ± 0.80	0.41	0.72 ± 0.80	0.37	0.26	−3.19 ± 1.02	**0.002**	−3.41 ± 1.00	**<0.001**	<0.001
Fat mass (%)	2.25 ± 1.23	0.068	2.18 ± 1.23	0.078	0.33	−4.94 ± 1.57	**0.002**	−4.64 ± 1.54	**0.003**	<0.001
Fat mass index (kg/m^2^)	2.37 ± 2.36	0.32	2.41 ± 2.36	0.31	0.28	−11.00 ± 2.98	**<0.001**	−11.15 ± 2.92	**<0.001**	<0.001
Fat mass to fat-free mass ratio	99.06 ± 46.96	**0.036**	96.41 ± 47.04	**0.041**	0.34	−197.30 ± 59.99	**0.001**	−185.61 ± 59.01	**0.002**	<0.001
24 h milk production parameters										
24 h milk production (g)	−0.28 ± 0.03	**<0.001**	−0.28 ± 0.03	**<0.001**	0.62	0.47 ± 0.03	**<0.001**	0.46 ± 0.03	**<0.001**	0.049
Breastfeeding frequency ^7^	−8.45 ± 1.79	**<0.001**	−8.37 ± 1.79	**<0.001**	0.40	0.38 ± 2.42	0.87	−0.11 ± 2.38	0.96	<0.001
Expressing frequency ^7^	6.79 ± 2.19	**0.002**	6.60 ± 2.21	**0.003**	0.47	0.95 ± 2.92	0.75	2.21 ± 0.77	0.44	<0.001
Milk removal frequency ^7^	−4.42 ± 2.00	**0.028**	−4.53 ± 2.00	**0.025**	0.24	1.20 ± 2.61	0.65	1.62 ± 2.56	0.53	<0.001
Mean breast storage capacity (g) ^8^	−0.78 ± 0.13	**<0.001**	−0.77 ± 0.14	**<0.001**	0.58	1.45 ± 0.20	**<0.001**	1.37 ± 0.20	**<0.001**	0.063
Mean PAMR (%) ^9^	−0.04 ± 0.63	0.95	−0.004 ± 0.62	0.99	0.12	0.27 ± 0.95	0.77	0.17 ± 0.93	0.85	0.004
Maternal expressing status										
Expressing status	NA	**<0.001**	NA	**<0.001**	0.57	NA	0.45	NA	0.44	<0.001
EP ^10^ vs. BF ^11^	147.83 ± 35.36 ^3^	**<0.001**	147.01 ± 35.43	**<0.001**	0.57	NA	NA	NA	NA	NA
OP ^12^ vs. BF ^11^	−0.35 ± 17.15	1.00	−0.44 ± 17.17	1.00	0.57	NA	NA	NA	NA	NA
PP ^13^ vs. BF ^11^	117.67 ± 29.03	**<0.001**	115.91 ± 29.23	**<0.001**	0.57	NA	NA	NA	NA	NA
OP ^12^ vs. EP ^10^	−148.17 ± 37.02	**<0.001**	−147.45 ± 37.09	**<0.001**	0.57	NA	NA	NA	NA	NA
PP ^13^ vs. EP ^10^	−30.16 ± 43.81	0.89	−31.10 ± 43.89	0.89	0.57	NA	NA	NA	NA	NA
PP ^13^ vs. OP ^12^	118.01 ± 31.04	**<0.001**	116.35 ± 31.21	**0.001**	0.57	NA	NA	NA	NA	NA

Data are parameter estimate (PE) ± standard error of estimate (SE); effects of predictors taken from ^1^ univariable linear models and ^2^ linear models that accounted for birth weight and ^3^ Tukey’s all-pair comparisons. Bold font indicates significant result. ^4^ Includes infant intake of breast milk from breast, expressed breast milk and formula. ^5^ *n* = 278; ^6^ *n* = 243. ^7^ Frequencies from both breasts combined. ^8^ *n* = 201; ^9^ *n* = 199; ^10^ *n* = 14; ^11^ *n* = 174; ^12^ *n* = 73; ^13^ *n* = 20. BF, breastfeeding; EP, exclusively pumping; NA, not applicable; OP, occasionally pumping; PP, predominantly pumping.

**Table A3:** Standardised regression estimates and fit statistics for mediation model predicting milk production.

Mediation Effects	Standardised Regression Estimate (Standard Error)	*p*-Value	Fit Statistics ^1^	
Total	−0.19 (0.05)	<0.001	CFI:	0.98
Direct	−0.06 (0.04)	0.15	TLI:	0.95
Indirect	−0.13 (0.04)	<0.001	RMSEA:	0.05
%Mediated	67.5%		SRMR:	0.04

^1^ Fit statistics: Goodness of fit statistics (CFI, Comparative Fit Index; TLI, Tucker–Lewis Index) > 0.90 indicate a good fit; badness-of-fit statistics (RMSEA, root mean squared error of approximation; SRMR, standardised root mean squared residual) < 0.08 indicate a good fit.
